# Crossing Total Occlusions Using a Hydraulic Pressure Wave: Development of the *Wave* Catheter

**DOI:** 10.3389/fmedt.2022.851927

**Published:** 2022-04-01

**Authors:** Aimee Sakes, Menno Lageweg, Remi I. B. van Starkenburg, Saurabh Sontakke, Jo W. Spronck

**Affiliations:** ^1^Department of BioMechanical Engineering, Faculty of Mechanical, Maritime, and Materials Engineering, Delft University of Technology, Delft, Netherlands; ^2^Department of Electronic and Mechanical Support Division (DEMO), Delft University of Technology, Delft, Netherlands; ^3^Department of Precision and Microsystems Engineering, Faculty of Mechanical, Maritime, and Materials Engineering, Delft University of Technology, Delft, Netherlands

**Keywords:** buckling prevention, catheter technology, hydraulic pressure wave, medical device design, Minimally Invasive Surgery (MIS), Percutaneous Coronary Intervention (PCI)

## Abstract

With the ongoing miniaturization of surgical instruments, the ability to apply large forces on tissues for resection becomes challenging and the risk of buckling becomes more real. In an effort to allow for high force application in slender instruments, in this study, we have investigated using a hydraulic pressure wave (COMSOL model) and developed an innovative 5F cardiac catheter (*L* = 1,000 mm) that allows for applying high forces up to 9.0 ± 0.2 N on target tissues without buckling. The catheter uses high-speed pressure waves to transfer high-force impulses through a slender flexible shaft consisted of a flat wire coil, a double braid, and a nylon outer coating. The handle allows for single-handed operation of the catheter with easy adjusting of the input impulse characteristic, including frequency (1–10 Hz), time and number of strokes using a solenoid actuator, and easy connection of an off-the-shelf inflator for catheter filling. In a proof-of-principle experiment, we illustrated that the Wave catheter was able to penetrate a phantom model of a coronary Chronic Total Occlusion (CTO) manufactured out of hydroxyapatite and gelatin. It was found that the time until puncture decreased from 80 ± 5.4 s to 7.8 ± 0.4 s, for a stroke frequency of 1–10 Hz, respectively. The number of strikes until puncture was approximately constant at 80 ± 5.4, 76.7 ± 2.6, and 77.7 ± 3.9 for the different stroke frequencies. With the development of the *Wave* catheter, first steps have been made toward high force application through slender shafts.

## Introduction

### The Challenge of Minimally Invasive Surgery

Following the introduction of laparoscopic cholecystectomy by Mouret in France (1), the use of minimally invasive approaches has rapidly changed the performance of surgical procedures in a wide range of surgical specialties, including general surgery, urology, gynecology, thoracic surgery, plastic surgery, and cardiac surgery (2–4). In MIS, long and slender instruments are used to perform the procedure. These slender instruments replace the standard surgical tools and the surgeon's hands and eyes in performing different tasks, such as cutting, manipulating, and compressing tissues.

The revolution from conventional open surgery to Minimally Invasive Surgery (MIS) has been fueled by the desire to perform surgical procedures while avoiding the morbidity of conventional surgical wounds (5). Even today, the pain, discomfort, and disability, or other morbidity occurring as a result of surgery are more frequently due to the trauma involved in gaining access to the area to perform the intended procedure, rather than to the procedure itself (2, 3). It is, therefore, imperative that in future the damage to healthy tissues is minimized even further. In an effort to reduce the incision sizes and number of incisions even further, new MIS approaches, such as Natural Orifice Transluminal Endoscopic Surgery (NOTES) and Single-Incision Laparoscopic Surgery (SILS), and accompanying instruments are currently being developed.

### Buckling Prevention of Slender Tools

Unfortunately, with the use of slender instruments, the ability to exert high forces to, for example, resect tissues, becomes challenging. Slender instruments, such as needles, guidewires and catheters, become more prone to buckling, and thus failure, when applying high axial loads. Buckling, in turn, will lower the amount of axial force that can be applied by the instrument, as well as potential harm surrounding structures due to the sudden lateral deflection of the shaft.

In order to achieve high force application through slender tools, buckling needs to be prevented. Especially, when anticipating further miniaturization of surgical equipment in future. As of now, achieving tissue damage to effectuate resection is often achieved by using ultra sharp tips, such is seen in scalpels and needles. This will lower the required force to cut through tissues and, therefore, reduces the chance of buckling. However, sharp tips are ineffective when trying to cut or fragment brittle or hard tissues, such as bone, kidney- or gallstones, as the cutting edge will deteriorate rapidly. In these operations, blunt tips are often used. For these types of tissues, other buckling prevention strategies should be investigated.

In a previous study by Sakes et al. (6), different buckling prevention strategies used in nature were reviewed. It was found that you can make a distinction between static buckling prevention strategies—in which the geometry of the tool is optimized—and dynamic buckling prevention strategies—in which the loading characteristic is altered. In static buckling prevention strategies, buckling of slender instruments, such as catheters and needles, can in theory be prevented by decreasing their slenderness λ—defined as the length *L* (mm) divided by the diameter Ø (mm) of the instrument—, by increasing the Young's Modulus *E* (GPa), or by increasing the second moment of area *I* (mm^4^) (6, 7). However, in an effort to reduce harm to the patient and reach deep inside the human body, the instrument diameter is expected to decrease further in future. Furthermore, as more and more locations can be reached inside the human body, the length of the devices is expected to increase. Therefore, the geometry of the instruments cannot be altered significantly in order to improve the buckling resistance and the buckling resistance is even expected to go down in future.

With this trend of further miniaturization of medical instruments in mind, dynamic buckling prevention strategies are advantageous over static buckling prevention strategies, as these strategies allow for increasing the critical buckling load of slender tools independent from their geometrical features. In dynamic buckling prevention strategies, a dynamic load is used to increase the critical buckling load of the tool. The dynamic load is usually an impulse, which is defined by a force $\vec{F}$ (N) acting for the time interval *t* (s), or a (high-frequency) vibratory input $\vec{F}=F_{0}\sin\omega t\left[N\right]$. In dynamic buckling, the critical buckling load can far exceed the static critical buckling load, as long as the load is removed fast enough. This is mainly due to the fact that some time is required for transition from an unstable straight configuration to a stable bend configuration (8). This time is inversely proportional to the cubic root of the compression velocity of the tool's end (9). Maximum buckling occurs near the impact end at a wavelength much shorter than the length of the rod, and at a stress many times the buckling stress of a statically-loaded tool. Furthermore, using an impulse to dynamically load the catheter does not only prevent buckling by increasing the critical load, it also has been shown to lower the required penetration load, as illustrated in the studies of Heverly et al. and Jelínek et al. (10, 11), as the environmental damping and the inertia of the target tissue can act as a reaction force to the impulse force.

### Transferring High Force Impulses

Transferring high force impulses through a flexible tube is challenging. In order to apply an impulse *J* onto the target tissue, translational momentum *p*, defined as the product of mass *m* (kg) and velocity *v* (m/s), should be generated inside the catheter. Translational momentum can be generated either locally at the distal tip or proximally in the handle. Subsequently, the translational momentum should be transferred to the target location. Whereas, in rigid instruments, the shaft itself is able to transfer the momentum directly to the target area with high efficiency, in flexible systems significant energy loss will be incurred due to energy dissipation. In order to transfer impulse through a slender flexible shaft with high efficiency, you need an incompressible medium, such as a (flexible) rod, a series of rigid elements, or a hydraulic fluid; an axially and radially stiff shaft; and prevent wave reflection inside the system. In this study, we propose to use a hydraulic pressure wave to transfer translational momentum through the catheter.

### Goal of This Study

The goal of this study is to develop a slender flexible instrument that can exert high force [>1.5 *N* (12)] impulses on the target tissue without buckling. This instrument can be used for a variety of purposes and operations; however, we will use the Percutaneous Coronary Interventions (PCIs) of Chronic Total Occlusions (CTOs) as our example case to build on. PCIs of CTOs are considered the last frontiers of cardiovascular interventions and represent one of the most challenges cases due to the diameter restriction of Ø1.$\bar{6}$-2.$\bar{3}$ mm (5–7F), flexibility requirement, and the need for high force transfer [>1.5 *N* (12)]. The main failure mode in PCIs of CTOs is the inability to cross the occlusion, accounting for ~60% of the failure cases (13). The inability to cross is a result of guidewire buckling, as the force needed to puncture the CTO often exceeds the critical buckling load (*F*_*critical*_ [*N*]) of the guidewire, which is around 0.008–0.26 N depending on the type of guidewire (14).

### Current State-of-the-Art in CTO Crossing

Current tools for crossing CTOs during PCIs range from dedicated guidewires and catheters to dedicated resection devices (14, 15). Dedicated guidewires, such as the ones in the *Miracle* series, *Conquest* series, and *Confiamza* series from Asahi Intecc, have been specifically developed to improve PCIs of CTOs (16). These dedicated guidewires usually have higher tip loads than conventional guidewires, which allow them to apply higher forces on the CTO, and are often used in conjunction with specialized micro-catheters, such as *Tornus* (Asahi Intecc), *Corsair* (Asahi Intecc) and *Crossboss* (Boston Scientific) to improve guidewire support during crossing (17). Unfortunately, even with these advancements, crossing CTOs remain challenging, substantiated by the relatively low success rate of about 80% in comparison to acute occlusions (18). For this purpose, specialized resection devices, such as the rotational atherectomy device *Rotablator* (Boston Scientific) and *Excimer Laser Systems* have been developed (16). These devices are infrequently used during CTO PCI and are associated with similar technical success than the use of specialized guidewire and catheter combinations, but with a higher risk for donor vessel injury and tamponade requiring pericardiocentesis (i.e., a procedure to remove fluid buildup in the pericardium) (19). Furthermore, if it is impossible to cross the CTO directly, a reentry approach is considered using the *Stingray catheter* (Boston Scientific) or *ReCross Device* (IMDS), for example (16, 17). These devices allow for bypassing the CTO through the blood vessel wall and subsequently the creation of a new lumen. The downside of this technology is that considerable damage is done to the blood vessel wall, which can increase the risk of perforation and distal hematoma (20). In this study we propose to develop a device that can apply high forces in order to cross occlusions through the true lumen, but is not associated with the increased risk of complications, like the specialized resection devices.

### Layout of This Study

In the upcoming section, we will describe the proposed solution. Subsequently, we will describe the developed finite element multiphysics COMSOL model. This COMSOL model was developed in order to determine the effect of input pressure, internal roughness, shaft curvature, and fluid viscosity, on the efficiency of the system. Secondly, based on the results of the COMSOL model, the design process and experiments of the catheter shaft will be discussed, leading up the final design of the catheter. In Section Proof of Principle Experiment, the proof of principle experiments in a 3D-printed circumflex artery will be discussed. Followed by the discussion and conclusion in Sections Discussion and Conclusion, respectively.

## Proposed Solution

**Figure 1:**
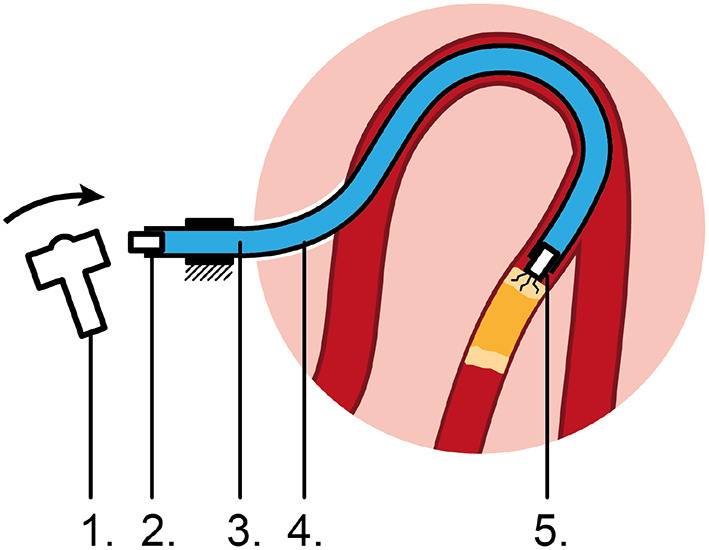
Proposed flexible impulse hydraulic catheter. 1: Input mechanism, 2: Input piston, 3: Hydraulic medium, 4: Flexible catheter shaft, 5: Output piston impacting on the CTO, causing micro cracks.

In order to transfer high-force (>1.5 *N*) impulses, we propose to use a hydraulic medium. In its simplest form, the concept consists of the following five elements (Figure 1): (1) an input mechanism at the handle, (2) an input piston, (3) a hydraulic fluid as medium, (4) a flexible catheter shaft, and (5) an output piston at distal tip of instrument. In order to initiate translational moment in the fluid, a sudden chance in velocity of the fluid is needed. The input mechanism is shown here as a hammer impacting the input piston to create an input impulse *J*_*input*_ (Ns). The input mechanism can, however, have many forms to create the required input signal. The applied input impulse is subsequently converted into translational momentum *p* of a fluid in the form of a hydraulic pressure wave; a longitudinal wave with regions of increased density, known as compressions, and regions of reduced density, known as rarefactions, through a fluid column. A common example of a hydraulic pressure wave is the water hammer, which can occur when a fluid in motion is suddenly forced to stop by a suddenly closed valve, such as a water tap. Finally, the translational momentum *p* of the fluid is converted into an output impulse *J*_*output*_ (Ns).

In a previous study, we have determined the feasibility of using a hydraulic pressure wave to transfer high-force impulses over a distance of 1 m through 6F commercially available catheters (21). In this study, an experiment was performed in which the input and output impulses were recorded as a function of the catheter type, curvature, and output plunger travel distance. It was observed that a higher input impulse and a more flexible catheter design negatively influenced the efficiency, whereas catheter curvature did not have a significant effect. Furthermore, we were able to transfer a high-force impulse of 40.5 N without buckling (21). The ability to transfer high-force impulses through a highly tortuous shaft and under angle, allows for effective treatment of CTOs at any location inside the human body, such as the coronary arteries and the extremities. In this study, we will investigate which shaft, input, and fluid parameters are of importance to transfer the high-force impulse from the handle toward to tip using COMSOL. Subsequently, we will use these insights to develop a dedicated catheter shaft and handle that can be used in a (close to) clinical setting in future.

## Design Process

### Design Parameterization

#### Catheter COMSOL Model Design

##### COMSOL Model

In an effort to better understand the transmission losses of the hydraulic pressure wave, a finite element multiphysics COMSOL model was constructed. For this purpose, we used the water hammer interface in COMSOL, as the physics involved in our proposed solution is analogous to the water hammer phenomenon. In this phenomenon, there is a two-way coupling of the fluid and solid mechanics domains. This renders the problem analytically and computationally complex. In order to solve this challenge, the water hammer interface in COMSOL uses the continuity equation and the momentum equations for a compressible fluid traveling inside pipes of variable cross section.

The mass conservation for a fluid inside a pipe is represented by the following formula:


(1) $$\begin{array}{l} \frac{\partial A_{\rho }}{\partial t}+\nabla \cdot \left(A\rho u\right)=0 \end{array}$$

where *A* is the cross-sectional area of the pipe, ρ is the fluid density, and *u* is the tangential fluid velocity. For adiabatic processes, the density and cross section area are functions of the pressure, so the continuity equation looks like shown below:


(2) $$\begin{array}{l} \frac{\partial A\left(p\right)\rho \left(p\right)}{\partial t}+\nabla \cdot \left(A\left(p\right)\rho \left(p\right)u\right)=0 \end{array}$$

In a first order approximation, this can be represented by the following formula:


(3) $$\begin{array}{l} A_{0}\left(\frac{1}{K_{\rho }}+\frac{1}{K_{A}}\right)\rho_{0}\frac{\partial p}{\partial t}+\nabla \cdot \left(A_{0}\rho_{0}u\right)=0 \end{array}$$

where, *K*_ρ_ is the bulk modulus of the fluid, and *K*_*A*_ is the effective bulk modulus of the cross-sectional area. *A*_0_ and ρ_0_ are the reference area and reference density at a given pressure *p*_0_. The water hammer wave speed *c*, in a pipe with zero axial stresses, is calculated using a combination of fluid and structural properties. It is given by the following equation known as the Korteweg formula (22):


(4) $$\begin{array}{l} \frac{1}{c^{2}}=\rho_{0}\left(\frac{1}{K_{\rho }}+\frac{1}{K_{A}}\right) \end{array}$$

The effective bulk modulus for the cross-sectional area *K*_A_ is given by the pipe's material properties in the following manner:


(5) $$\begin{array}{l} K_{A}=E\frac{w}{d_{h}} \end{array}$$

where, *E* is the Young's modulus, *d*_h_ is the hydraulic diameter, and *w* is the pipe's wall thickness. For a pipe with a circular cross-section, as is the case in this project, the hydraulic diameter is equal to the inner diameter of the pipe. The momentum equation is written as follows:


(6) $$\begin{array}{l} \rho \frac{\partial u}{\partial t}=-\nabla p-f_{D}\frac{\rho }{2d_{h}}u|u|+F \end{array}$$

where, *F* are the body forces on the fluid, *f*_D_ is the Darcy friction factor, which is normally a function of the Reynolds number, the surface roughness and the hydraulic diameter. From the above equations, a set of equations that are generally known as the water hammer equations are obtained and are written as follows:


(7) $$\begin{array}{l} A\frac{1}{c^{2}}\frac{\partial p}{\partial t}+\nabla \cdot \left(A\rho u\right)=0 \end{array}$$

(8) $$\begin{array}{l} \rho \frac{\partial u}{\partial t}=-\nabla p-f_{D}\frac{\rho }{2d_{h}}u|u|+\rho g \end{array}$$

where the body force is represented by the gravity force *g*.

Based on these equations, it becomes clear that both the input, catheter and fluid parameters, such as Young's modulus *E*, wall thickness *w*, the hydraulic diameter *d*_*h*_, surface roughness (as part of *f*_*D*_), the fluid pressure *p*, and bulk modulus *K*_ρ_ of the fluid (amongst others) are of importance for wave propagation. For application of this phenomenon in a catheter, we set out to investigate these parameters using a simplified catheter model, with the exception of the wall thickness and hydraulic diameter. Since we are bound by a maximum outer diameter, due to size constraints set by the vasculature, and minimum inner diameter, due to size constraints set by the guidewire diameters, the wall thickness is set to 0.15 mm and the lumen diameter to 1.4 mm. Furthermore, for clinical application, we also wanted to investigate the effect of shaft curvature on the efficiency.

**Figure 2:**
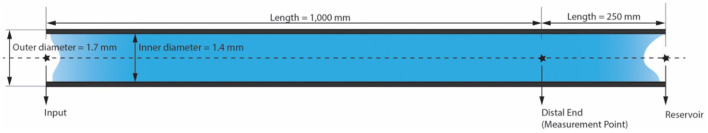
Sketch of COMSOL model of the catheter shaft filled with a fluid.

Two main scenarios were analyzed: (1) a stainless-steel shaft and (2) a PTFE shaft. The shaft, which represents the catheter, is assumed to have a circular cross-section of the same diameter throughout its length. It is assumed to be anchored at the proximal end, while being free to move at the distal end (allowing for bending and rotational motions of the tip). This is similar to the clinical situation, in which the distal end of the catheter (the tip) is free to move sideways, whereas the handle is kept in place by the interventionists. The Young's modulus and the Poisson's ratio of the pipe material are changed according to the material used for the simulation. The length of the shaft (*L*) is 1 250 mm and the distance of the measurement point from the reservoir (*z*; where the impulse is created) is 250 mm, which makes the length of the shaft that represents the catheter 1,000 mm. The inner diameter of the pipe (*d*) is 1.4 mm and the thickness of the pipe wall (*w*) is 0.15 mm. The catheter is modeled as a 2D structure (see Figure 2). Initial pressure (*p*) was set to atmospheric pressure. A user defined mesh is implemented by choosing the number of elements (*N*) to be 100. Each element is a line segment of length *dx* along the pipe length. Thus, *dx* = *L*/*N* = 12.5 mm.

The system was simulated with two input pressures (*p* = 1 and 15 MPa), two internal roughness values (1.5 and 15 μm), three different values of viscosity of the medium (μ = 1.0, 3.0, and 5.0 mPa·s), and two different curvatures of the shaft (2 × 90° and 4 × 90° curves). Efficiency of the system is expressed as the ratio of the output pressure pulse amplitude to the input pressure pulse amplitude (see Table 1).

**Table 1:** COMSOL model results.

**Shaft material**	**Input (MPa)**	**Roughness (μm)**	**Output (MPa)**	**Efficiency (%)**	**Wave velocity (m/s)**
PTFE	1	1.5	0.88	88	230.2
PTFE	1	15	0.86	86	230.2
PTFE	15	1.5	11	73	230.2
PTFE	15	15	6.8	45	230.2
Stainless steel	1	1.5	1	100	1,426.8
Stainless steel	1	15	1	100	1,426.8
Stainless steel	15	1.5	14.8	98	1,426.8
Stainless steel	15	15	14.5	97	1,426.8

*The configuration with the highest efficiency is indicated in green. The PTFE configuration with the highest efficiency is indicated in blue*.

##### Input Pressure

**Figure 3:**
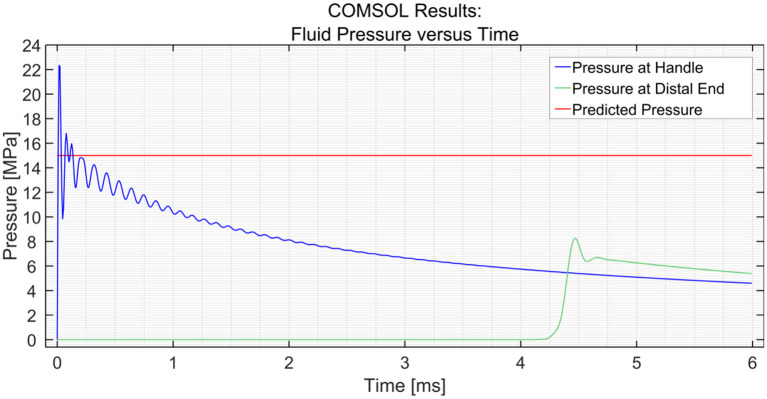
Pressure variation at the input and the distal end vs. time in the PTFE shaft. The input pressure was set to 15 MPa.

Figure 3 shows the dynamic pressure of the PTFE shaft at 2 measurement points; the input (handle) and the distal end. It can be seen that the pressure at the input increases almost immediately to 22 MPa at *t* = 0 ms, yet soon stabilizes around 15 MPa. The pressure drops as the pulse moves along the shaft toward the distal end, which is 1,000 mm away from the input. The pressure wave reaches the measurement point at time *t* = 4.3 ms, resulting in a wave velocity of ~230 m/s. The efficiency of the hydraulic pressure wave is calculated by taking the average value of the oscillation of the input (15 MPa) and output value (6.8 MPa) and is found to be ~45%. In the case of a stainless-steel pipe, the efficiency is 100% when the input is a pressure pulse of (1 MPa) and even if it is increased to (15 MPa), the efficiency is almost 100%. This means that the losses due to the Darcy friction factor are less when working with stainless steel as compared to those when working with PTFE.

##### Internal Roughness

In an effort to research the effect of internal roughness of the wall, the roughness was changed from 1.5 to 15 μm. It was found that the roughness had a significant effect on the efficiency in the PTFE shaft with a 15 MPa input, whilst no significant effect was illustrated in the stainless-steel shaft (Table 1).

##### Viscosity of the Medium

**Figure 4:**
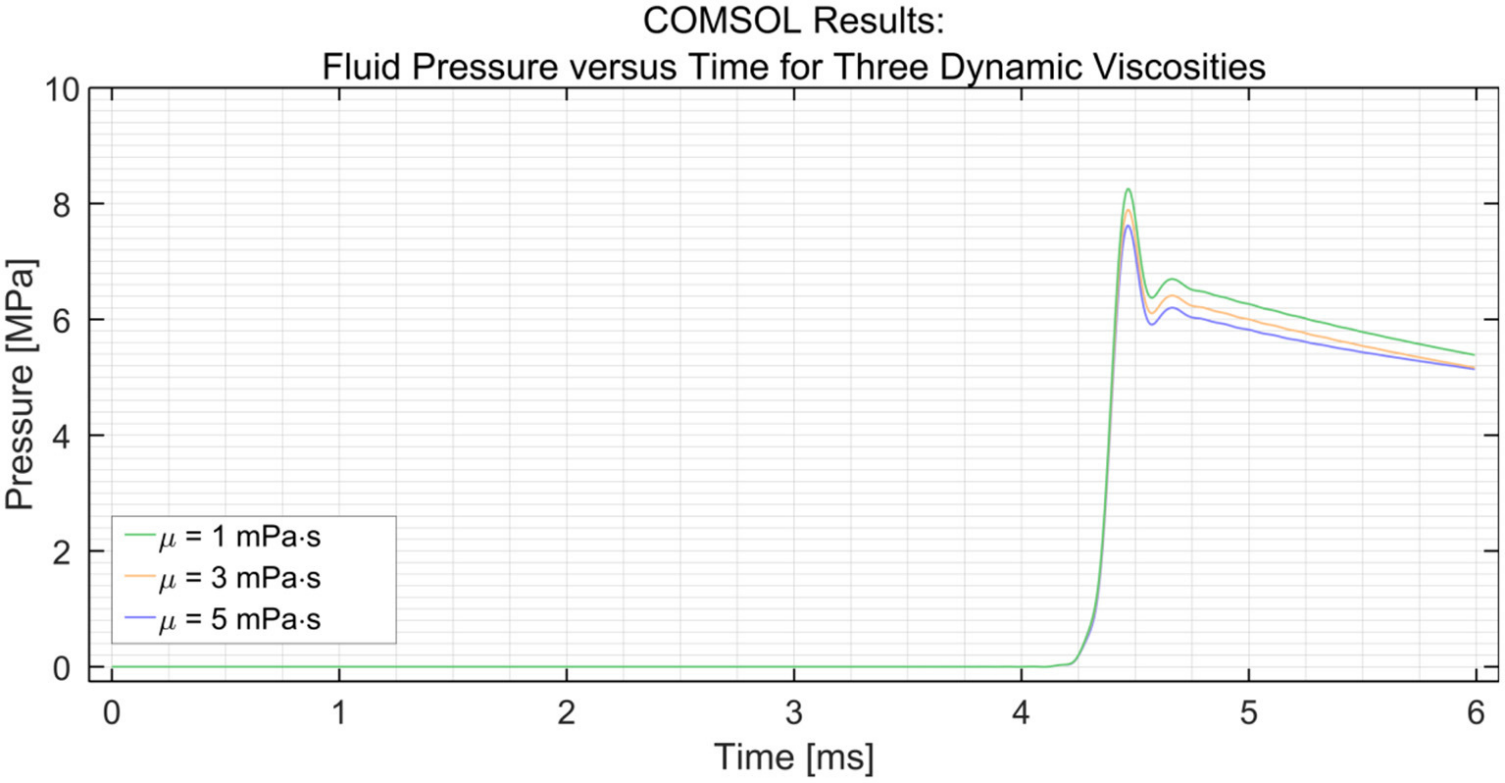
Pressure variation at the distal end vs. time in the PTFE Shaft for three dynamic viscosities (μ) of the medium; 1, 3, and 5 mPa·s. The input pressure was set to 15 MPa.

Using the parametric sweep tool in COMSOL, the effect of the dynamic viscosity of the medium on the efficiency was researched (see Figure 4). The dynamic viscosity (μ) of water at 20°C is 1 centipoise or 1 mPa·s (23). The dynamic viscosity of saline at 20°C is about 1.02 mPa·s at 0.9 wt% sodium chloride concentration (24). It can be seen that the efficiency decreases with an increase in dynamic viscosity. However, the change in efficiency is minimal; a drop of only 0.4 MPa is observed with a 300% increase in dynamic viscosity between 1 and 3 mPa·s. A similar drop in pressure of 0.4 MPa is observed between 3 and 5 MPa.

##### Curvature of the Shaft

**Figure 5:**
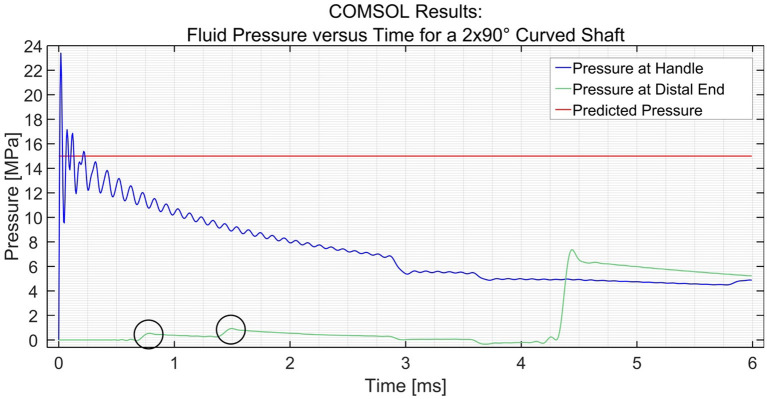
Pressure variation at the distal end and input vs. time in the PTFE Shaft for a shaft with 2 × 90° curves. The input pressure was set to 15 MPa. The black circles indicate the two curves.

**Figure 6:**
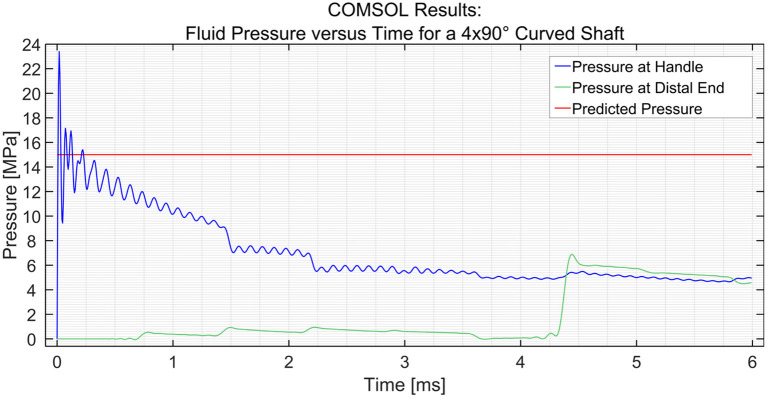
Pressure variation at the distal end and input vs. time in the PTFE Shaft for a shaft with 4 × 90° curves. The input pressure was set to 15 MPa.

In order to simulate the effect of catheter curvature, two curves of 90° were introduced at 1/6th and 2/6th of the length of the shaft between the input and the distal end. The two black circles shown in Figure 5 show an interesting phenomenon experienced by the distal end. If the shaft is straight throughout, as was the case in the previous situation, the pressure at the distal end remains zero until the hydraulic pressure wave travels to that point. However, when two 90° curves with a radius of 100 mm are introduced in the shaft, the losses that are introduced in the model act as small step inputs. At each bend, a positive pulse and a negative pulse originate inside the catheter shaft. The positive pulses travel toward the reservoir and the negative pulses travel toward the input. As the distal end is situated between the bends and the reservoir, it experiences 2 small pulses of pressure increase. The pulses travel further toward the reservoir and get reflected with 180° phase change. When they travel back toward the distal end, it experiences 2 negative pulses. This phenomenon does not have a significant effect on the efficiency of the system. Furthermore, even when four 90° bends were introduced (one full loop), the output pressure did not drop by a significant amount, as can be seen in Figure 6. The drop in pressure output observed when four curves were introduced was only 0.3 MPa.

#### Catheter Shaft and Tip Design

##### Catheter Shaft Design

In order to allow for quick adoption of the design in the PCI, it is imperative that the catheter is compatible with the PCI procedure and currently available instruments for PCI. Standard cardiac catheters used during PCIs of CTOs range between Ø1.$\bar{6}$-2.$\bar{3}$ mm (5–7F). However, in order to reach the most prevalent location of a CTO in the Right Coronary Artery (RCA) (25), an outer diameter of the catheter of Ø1.$\bar{6}$ mm (5F) and length of 1 000 mm was chosen.

##### Catheter Shaft Configuration

Based on the results of the COMSOL model and previous experiments (21), it can be concluded that the stiffness of the shaft is of significant influence on the efficiency. The catheter shaft should be able to resist radial and axial expansion caused by the pressure wave surge, while still allowing for bending motion. In collaboration with Asahi Intecc Co. LTD. (Nagoya, Japan), we developed an innovative catheter shaft that incorporates high radial and axial stiffness, whilst maintaining low bending stiffness. In order to achieve this, the catheter was constructed out of 3 main parts: (1) a flat wire coil, (2) a double braid, and (3) a nylon outer coating. The flat wire coil gives the catheter its radial stiffness, whereas the braid adds both radial and axial stiffness. A nylon outer coating was added to fix the double braid in place, add axial and radial stiffness, and allow for smooth guidance through the vasculature. All the materials used are biocompatible and allow for gamma radiation sterilization.

**Figure 7:**
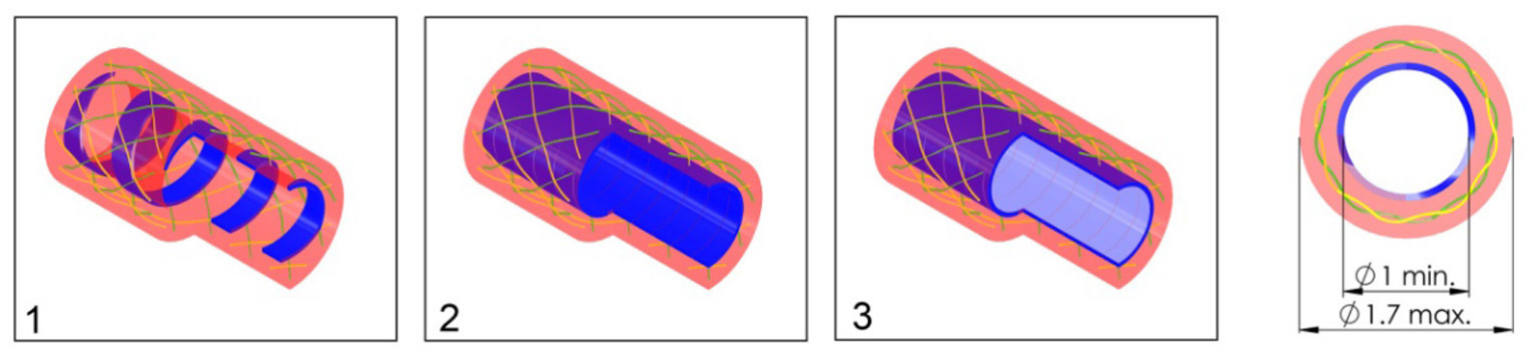
Catheter shaft design. From left to right: (1) isometric projection of the *open coil catheter shaft without PTFE liner*, (2) isometric projection of the *closed coil catheter shaft without PTFE liner*, (3) isometric projection of the *closed coil catheter shaft with PTFE liner*, and cross-sectional front view of the catheter shaft without PTFE liner. Color indications: blue = flat wire coil, green and yellow = double internal braiding, red = nylon outer coating, and light blue = PTFE liner.

In order to investigate the effect of the configuration of the shaft on the output force and impulse, three types of shafts were manufactured (see Figure 7):

1. A catheter shaft with an open flat wire coil without PTFE liner;
2. A catheter shaft with a closed flat wire coil without PTFE liner;
3. A catheter shaft with a closed flat wire coil with PTFE liner.

In the closed flat wire coil configuration, the coils of the spring-like structure touch, similar to an extension spring, whereas in the open flat wire coil configuration, the coils do not touch and there is a significant open space between them, similar to a compression spring. We hypothesized that the output force and impulse created by the pressure wave surge will be lower in the open flat wire coil shaft compared to the closed flat wire coil shaft, due to increased radial expansion of the catheter shaft in the latter configuration. Furthermore, the addition of a PTFE liner (wall thickness 0.01 mm) was investigated to substantiate if energy losses inside the hydraulic medium are partly due to the formation of turbulent flow vortexes and wave reflection by irregularities in the catheters' lumen. It was hypothesized that the addition of this PTFE liner, would decrease turbulent vortexes and reflections by smoothening the inner surface of the catheter and, therefore, increase the efficiency.

**Figure 8:**
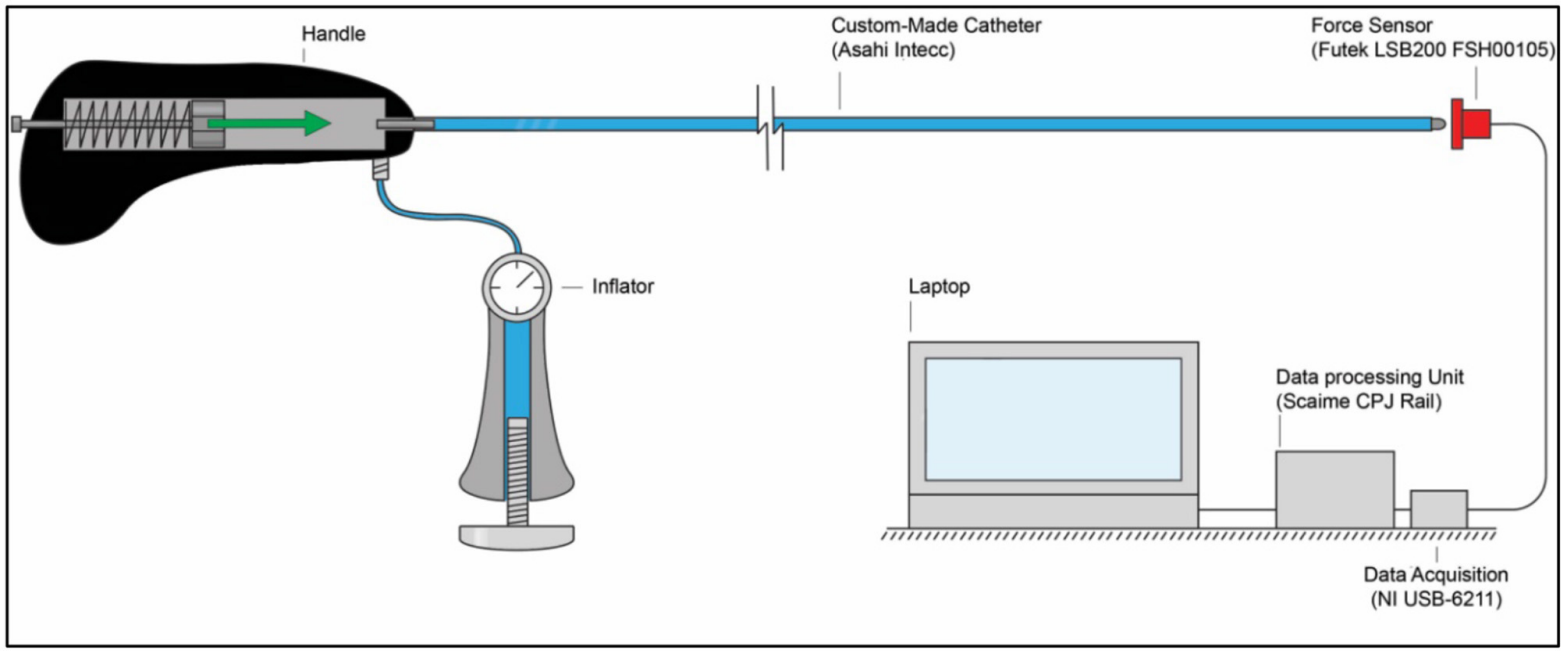
Experimental facility. The experimental facility consisted of the *Wave catheter* connected to the inflator, a force sensor (Futek LSB200 FSH00105), a data acquisition unit (National Instruments NI USB-6211), a data processing unit (Scaime CPJ Rail), and a laptop. The green arrow indicates the acceleration and motion of the mass-spring system inside the handle.

**Figure 9:**
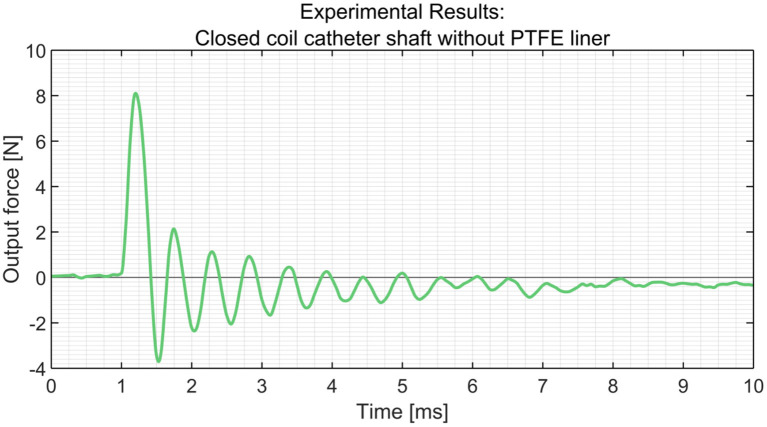
Experimental results obtained from the load cells using the closed coil catheter shaft without PTFE liner.

In order to measure the output impulse, the distal end of the catheter shaft was placed in contact with a load cell (*Futek LSB200 FSH00105*). A mass-spring system located inside the handle (on the proximal end of the catheter shaft) was used to generate the input impulse (see Figure 8). The output impulse was measured for all tests. Per measurement, the output impulse was determined by integrating the area under the force-time curve of the first peak (see also Figure 9). Each configuration was tested 3 times. An independent-samples *t*-test was conducted to compare the output impulse between the shaft with and the shaft without the PTFE liner, respectively.

**Figure 10:**
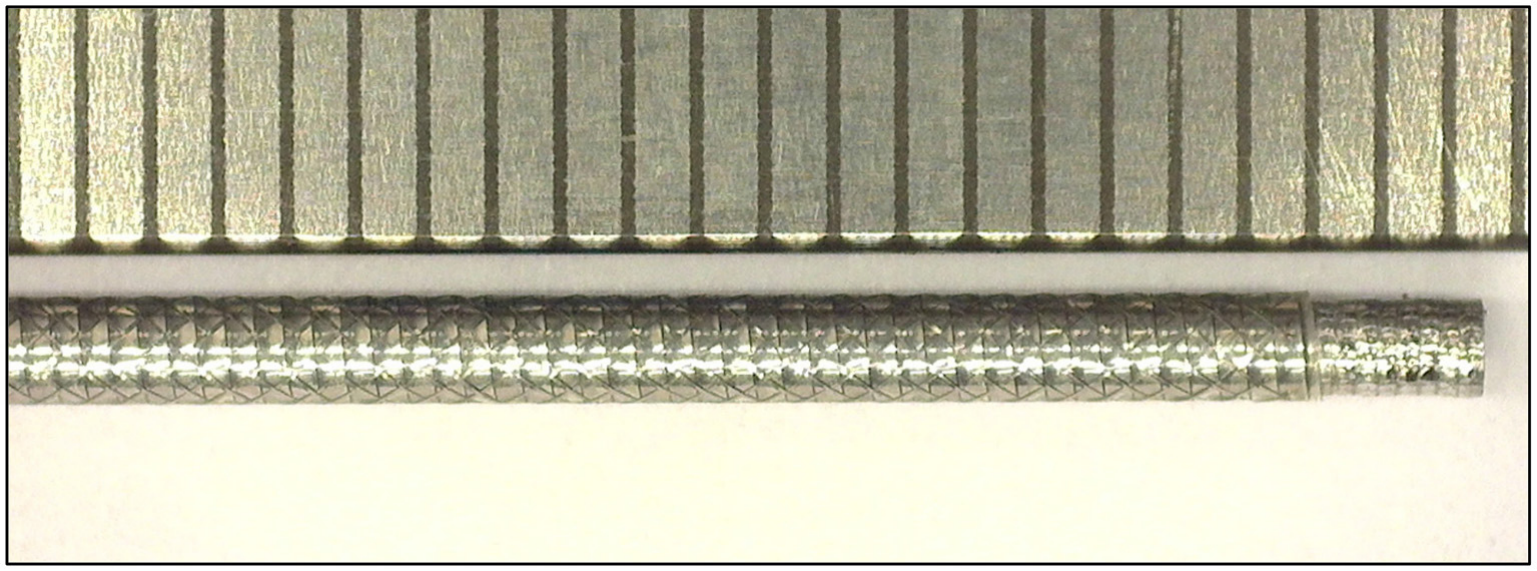
Close up of the final catheter shaft design. The ruler with 1 mm divisions is illustrated for scale purposes.

**Figure 11:**
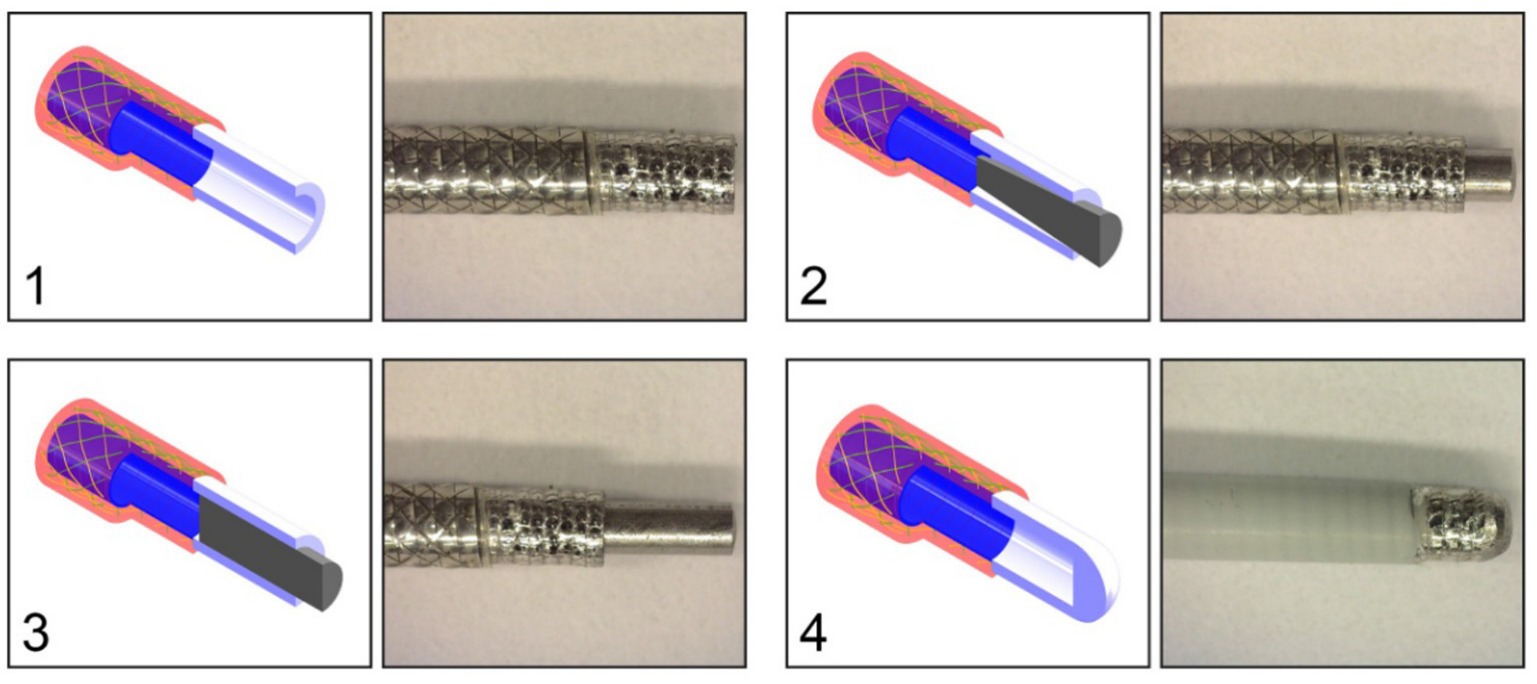
Catheter tip designs. Top left: (1) *open tip*. Top right: (2) *closed tip with conical pin*. Bottom left: (3) *closed tip with cylindrical pin*. Bottom right: (4) *closed compliant tip*. All the tips are connected to the *closed coil catheter shaft without PTFE liner*. Color indications: blue = flat wire coil, green and yellow = double internal braiding, red = nylon outer coating, white = tip, and gray = cylindrical or conical pin.

In Table 2, an overview of the test results is given. It was found that the *closed coil catheter shaft without PTFE liner* allowed for the highest output impulse. There was a statistically significant difference between the output impulse of the *closed coil catheter shaft without PTFE liner* with the *open coil catheter shaft without PTFE liner* (*p* = 0.0217) and *closed coil catheter shaft with PTFE liner* (*p* = 0.0220). These results confirm our hypothesis that more energy is dissipated in the *open coil catheter shaft with/without PTFE liner*. However, it was shown that the addition of the PTFE liner has a negative effect on efficiency, which is most likely due to energy dissipation by deformation of the liner. The subsequent experiments were performed using closed coil catheter shaft without PTFE liner (Figures 10, 11).

**Table 2:** Overview of the results (mean ± standard deviation, *n* = 3) of the catheter shaft type experiment.

**Catheter shaft type**	**Output force *F_***o***_* (*N*)**	**Output impulse *J_***o***_* (mNs)**
Open coil catheter shaft without PTFE Liner	1.6 ± 0.1	0.6 ± 0.1
Closed coil catheter shaft without PTFE Liner	7.7 ± 1.0	1.9 ± 0.3
Closed coil catheter shaft with PTFE Liner	6.0 ± 1.2	1.3 ± 0.3

*The catheter shaft type with the highest mean output force is indicated in green*.

##### Catheter Curvature

In order to substantiate the findings in the COMSOL model about the effect of curvature on the efficiency of the catheter, we subjected the closed coil catheter to a 360° loop with a radius of 100 mm. The experimental facility, protocol, and analysis was identical to the one described in the previous experiment, with the exception that the catheter shaft was in the looped configuration. A significant effect of curvature was found (*p* < 0.0001) according to the two-tailed *t*-test (see also Table 3).

**Table 3:** Overview of the results (mean ± standard deviation, *n* = 3) of the looped catheter experiment.

**Tip design**	**Output force F*_***o***_* (*N*)**	**Output impulse *J_***o***_* (mNs)**
Straight catheter configuration	7.7 ± 1.0	1.9 ± 0.3
Looped catheter configuration (360°, *r* = 100 mm)	1.0 ± 0.3	0.2 ± 0.1

*The catheter with the highest mean output force is indicated in green*.

##### Catheter Tip Design

At the distal end of the catheter, the hydraulic pressure wave's momentum should be transferred to an impulse. Slight movement of the distal end is necessary in order to transfer the impulse onto the target. In order to determine the most feasible and effective distal end design, four tips were tested: (1) *open tip*, (2) *closed tip with conical pin*, (3) *closed tip with a cylindrical pin*, (4) *closed compliant tip* (Figure 11). The experimental facility, protocol, and analysis was identical from that used in the catheter shaft configuration experiment. In Table 4, an overview of the experimental results is given. As can be seen, the *open tip design* resulted in the highest output force and impulse.

**Table 4:** Overview of the results (mean ± standard deviation, *n* = 3) of the tip design experiment.

**Tip design**	**Output force F*_***o***_* (*N*)**	**Output impulse *J_***o***_* (mNs)**
Open tip	7.7 ± 1.0	1.9 ± 0.3
Closed conical pin tip	6.3 ± 0.4	1.5 ± 0.2
Closed cylindrical pin tip	6.0 ± 0.8	1.3 ± 0.2
Closed compliant tip	2.8 ± 0.6	0.6 ± 0.2

*The tip design with the highest mean output force is indicated in green*.

##### Catheter Fluidic Medium

The impulse is transferred by ways of a hydraulic pressure wave toward the target. As illustrated in the COMSOL model, the dynamic viscosity of the fluid has a minor effect on the efficiency of the system. However, we hypothesize that energy losses in the fluidic medium are mainly the result of: (1) dissolved gas and (2) the compressibility of the fluidic medium itself. Gas in the system is caused by: (1) dissolved gas in the fluid and (2) gas inside the catheter while filling. In order to investigate the effect of dissolved gas inside the fluidic medium, three different types of still water were tested: *still water from the tap, still water rested (for 1 h)*, and *still water degassed*. In the still water degassed medium, all the dissolved gas was removed from the water. Furthermore, we also tested IV-fluid as a fluidic medium, which is a biocompatible fluid that can be used to fill the catheter in a clinical setting. In Table 5, an overview of the test results is given. As can be seen, the output force and impulse are largest for *still water degassed*.

**Table 5:** Overview of the results (mean ± standard deviation, *n* = 3) of the fluidic medium experiment.

**Tip design**	**Output force *F_***o***_* (*N*)**	**Output impulse *J_***o***_* (mNs)**
Still water from the tap	7.4 ± 0.6	1.8 ± 0.2
Still water rested	6.5 ± 0.6	1.3 ± 0.2
Still water degassed	7.7 ± 1.0	1.9 ± 0.3
IV-fluid	6.3 ± 0.3	1.7 ± 0.1

*The fluidic medium with the highest mean output force is indicated in green*.

#### Catheter Handle Design

**Figure 12:**
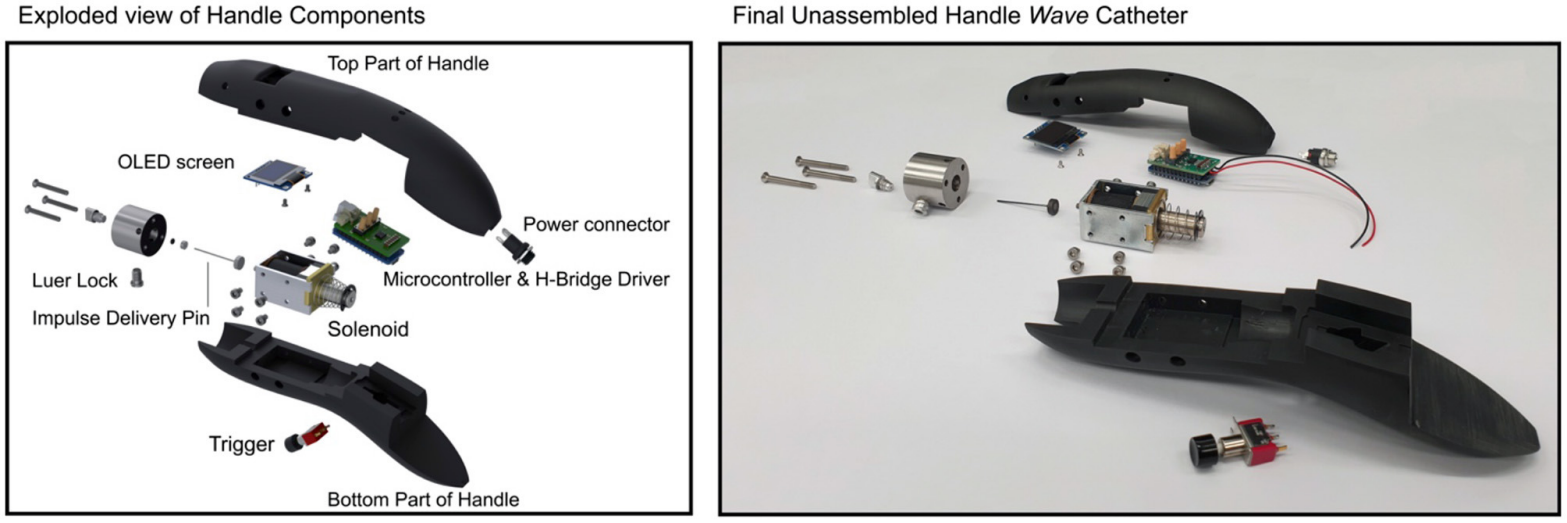
Exploded view of the final handle design.

In Figure 12, the final handle design is illustrated. The handle is designed to allow for easy one-handed control of the device. All controls for the functionalities are reachable by hand. Furthermore, its symmetric design allows for easy left- and right-handed use.

During PCIs of CTOs, inflators are used to inflate balloon catheters. In an effort to allow for easy integration in the PCI procedure, our developed catheter can be filled with the same inflator. The inflator can be filled with saline or IV-fluid and, subsequently, be connected to the handle using a Luer lock, after which it can be used to flush and fill the catheter before use.

The hydraulic pressure wave is generated inside the handle using a solenoid (Tremba HMA-2622d-0.002) in combination with a microcontroller (Arduino Pro Trinket 5 V, 16 MHz) and an H-Bridge driver. This solenoid is used to accelerate a Ø1 mm stainless steel impulse delivery pin. Wave reflection of the hydraulic pressure wave is minimized by placing the impulse delivery pen inside the catheter lumen. In order to prevent fluid leaking from the system, a Nitrile Butadiene Rubber (NBR) O-ring was placed around the impulse delivery pin.

The output force-time characteristic is adjustable to account for differences in biomechanical properties of CTOs. The output stroke is set (and limited) to 2 mm, in order to minimize the chance of blood vessel wall damage. An OLED screen in combination with three tactile buttons allow the user to select between three pre-set modes:

1. *Continuous mode*—In this mode, the solenoid is triggered continuously based on the set frequency (up to 10 Hz);
2. *Stroke mode*—In this mode, the preferred stroke count and frequency of the solenoid are selected;
3. *Time mode*—In this mode, the preferred time in seconds and frequency of the solenoid are selected.

After selecting the preferred mode and setting, the solenoid can be triggered using the trigger button on the bottom of the handle. Note that the acceleration of the impulse delivery pin of the solenoid, and therefore the applied force, is equal in each setting.

### Final Catheter Design

**Figure 13:**
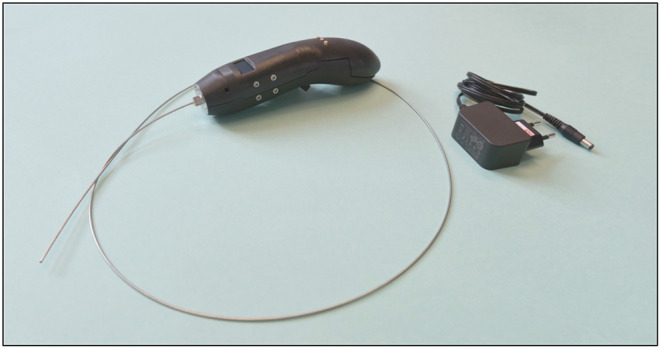
Final developed *Wave* catheter prototype.

In Figure 13, the final *Wave* catheter (5F, *L* = 1,000 mm, EI = 105 Nm^2^) is illustrated. The final *Wave* catheter consists of an inflator to fill the catheter, an electromechanical handle to generate a high-force impulse, the closed coil catheter shaft without PTFE liner to transfer the high-force impulse to the tip, an open tip to transfer the impulse onto the target.

## Proof of Principle Experiment

### Experimental Goal

The goal of the proof-of-principle experiment was to test the *Wave* catheter in a setting that closely resembles the clinical situation. As we use PCIs of CTOs as our test case, we will test our catheter using a CTO model located in one of the coronaries of the heart.

### Experimental Variables

#### Dependent Variables

In order to determine the effectiveness of the *Wave* Catheter for use during PCIs of CTOs, the following dependent variables were measured during the experiment:

- **Time until puncture** ***t***_***p***_**(s)**: In each experiment, the time until puncture was recorded. The time until puncture was defined as the time to fully cross, and thus penetrate, the CTO phantom model.
- **Number of strikes until puncture** ***n***_***p***_ **(#)**: In each experiment the number of strikes until puncture was calculated based on the time until puncture and the selected setting of the catheter.

#### Independent Variables

The *Wave* Catheter was tested for the following independent variable:

- **Stroke frequency** ***f***_***s***_ **(Hz)***:* The stroke frequency was set at 1, 5, and 10 Hz. The continuous mode of the solenoid was selected.

### Experimental Facility

The development of a representative CTO animal model has been proven difficult mainly due to the lack of spontaneous atherosclerosis in animals (26). Even though the use of animal models is preferred for device evaluation, these do not allow for consecutive tests and evaluation under constant test conditions. Therefore, it was decided to build an artificial CTO model. The CTO model was made from HydroxyApatite (HA) and a gelatin mixture (*sheet gelatin*, Dr. Oetker, Bielefeld, Germany). These materials were selected to mimic the high calcium and collagen concentrations found in the proximal caps of heavily calcified CTOs. As we focus on crossing the most calcified CTOs, we developed a CTO model consisting of a proximal cap (0.5 mm thick) with a HA content of 75 wt% and CTO body (10 mm long) with a HA content of 50 wt%. Whereas, reported average concentrations of calcium content within CTOs are generally lower than 75% (27), local calcium concentrations as high as 75% can be expected.

**Figure 14:**
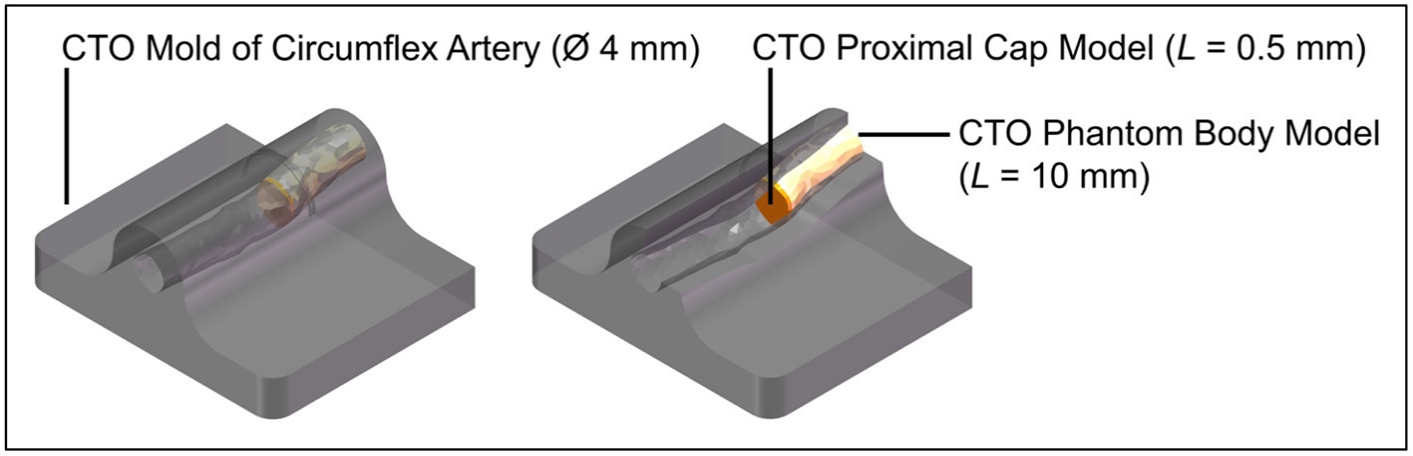
Experimental facility: CTO phantom model. Color indications: gray = 3D-printed mold of the circumflex artery, yellow = CTO phantom model body, and orange = CTO phantom model proximal cap.

The CTO model was poured into a 3D-printed (*Perfactory 4 Mini XL*, Envisiontec) negative mold of the circumflex artery that was obtained from the 3D-printed heart model (Materialise, Leuven, Belgium) (see Figure 14). After pouring the CTO models into the mold, they were refrigerated for 24 h, after which the experiment commenced. The whole model was surrounded by Blood-Mimicking Fluid (BMF) made of 25% weight percentage (wt) glycerine and 75 wt% clear water, to simulate blood with a viscosity between 2.5 and 2.8 mPa·s at 36–40°C (28).

### Experimental Protocol

At the beginning of the experiment, the CTO phantom model was placed inside a container filled with BMF. Subsequently, the Wave catheter was inserted into the container and CTO phantom model. The *Wave* catheter was guided toward the proximal cap by slowly pushing it through the model. Once arrived at the proximal cap, the *Wave* catheter was consecutively actuated in the continuous mode at 1, 5, and 10 Hz until puncture was observed into the CTO model. Each frequency was repeated three times.

### Data Analysis

The data obtained from the experiments were analyzed and processed using Matlab R2021a. Per condition the mean and standard deviation of the time until puncture and number of strikes until puncture were derived. A one-way ANOVA was executed to determine if there was a significant effect of the stroke frequency on the time until puncture and number of strikes until puncture.

### Experimental Results

In Table 6, the experimental results are indicated. The time until puncture was 80 ± 5.4 s, 15.3 ± 0.5 s, and 7.8 ± 0.4 s, for a stroke frequency of 1, 5, and 10 Hz, respectively. There was a significant difference between the time until puncture for the different stroke frequencies, according to a one-way ANOVA [*F*_(2, 6)_ = 0.35, *p* = 0.72]. The number of strikes until puncture was approximately constant at 80 ± 5.4, 76.7 ± 2.6, and 77.7 ± 3.9 for a stroke frequency of 1, 5, and 10 Hz, respectively. There was no significant difference between the number of strokes until puncture for the different stroke frequencies, according to a one-way ANOVA [*F*_(2, 6)_ = 325, *p* = 7.65·10^−7^].

**Table 6:** Experimental results (mean ± standard deviation, *n* = 3) of the proof-of-principle experiment.

**Selected frequency fs (Hz)**	**Time until puncture *t_***p***_* (s)**	**Number of strikes until puncture *n_***s***_* (#)**
1	80 ± 5.4	80 ± 5.4
5	15.3 ± 0.5	76.7 ± 2.6
10	7.8 ± 0.4	77.7 ± 3.9

## Discussion

### Summary of Main Findings

We have developed an innovative 5F cardiac catheter (*L* = 1,000 mm), called the *Wave* catheter, that allows for applying high forces up to 9.0 ± 0.2 N on target tissues, such as CTOs, without buckling. The catheter uses high-speed pressure waves to transfer high-force impulses through a slender flexible shaft consisting of a flat wire coil, a double braid, and a nylon outer coating. The distal end of the catheter is open, allowing for easy filling of the device and low risk of blood vessel perforation. The handle allows for single-handed operation of the catheter with easy adjusting of the input impulse characteristic, including frequency, and easy connection of an off-the-shelf inflator for catheter filling. The handle can be fitted to any cardiac catheter that is fitted with a Luer-lock at its proximal end, making the device very versatile. It must be noted that since the cardiac catheters are not optimized for transferring translational momentum, the choice of catheter will affect the efficiency of the system.

In our COMSOL model, we investigated the effect of input pressure, shaft elasticity, internal roughness, viscosity, and shaft curvature. It was found that the input pressure, shaft elasticity, internal roughness, and viscosity all had a significant effect on the efficiency of the catheter. This was in alignment with what was found in the shaft configuration experiments. For example, we found that the addition of a soft internal PTFE liner in the catheter shaft negatively affected the efficiency of our catheter due to energy dissipation in the system. There was, however, a discrepancy between the COMSOL modeling results and the actual catheter result with respect to the effect of curvature. In the COMSOL model, as well as our previous study (21), it was found that the curvature of the shaft does not have a significant effect on the efficiency of the system. In our experimental results with the developed catheter, we found that the curvature did have a significant effect. We hypothesize that this is due to the design of the catheter shaft itself. When placed into the curve, the closed flat wire coil might misalign slightly, which may cause irregularities in the lumen. This in turn may cause vortexes in the fluid that decreases the efficiency. We recommend that in a future version, the flat wire coil is opened slightly to give this structure space to bend. Furthermore, in order to increase the versatility of the *Wave* catheter, the ability to use clinically available catheters fitted to the developed handle to guide high force impulses, should be investigated.

In a proof-of-principle experiment, we illustrated that the *Wave* catheter was able to penetrate the CTO phantom model without displaying recoil. Recoil could potentially be harmful for the blood vessels, as sudden sideways deflection can result in high forces on the blood vessel wall. The phantom model was manufactured out of HA and gelatin and was made up of two specific regions representing the CTO proximal cap and body. It was found that the time until puncture decreased from 80 ± 5.4 s to 7.8 ± 0.4 s, for a stroke frequency of 1–10 Hz, respectively. The number of strikes until puncture was approximately constant at 80 ± 5.4, 76.7 ± 2.6, and 77.7 ± 3.9 for the different stroke frequencies. As can be seen from the experimental results, the energy required to penetrate the model is approximately the same for the 1, 5, and 10 Hz setting. We, therefore, recommend testing the *Wave* catheter at even higher frequencies to expedite the crossing procedure. The theoretical maximum frequency the *Wave* catheter can function is equal to the velocity of the pressure wave. In a previous study (21), we found that the fluid travels with an approximated velocity of 550–585 m/s through the catheter. This velocity translates into a maximum frequency of 550 Hz and thus an assumed crossing time of 0.14 s.

### Intended Use

**Figure 15:**
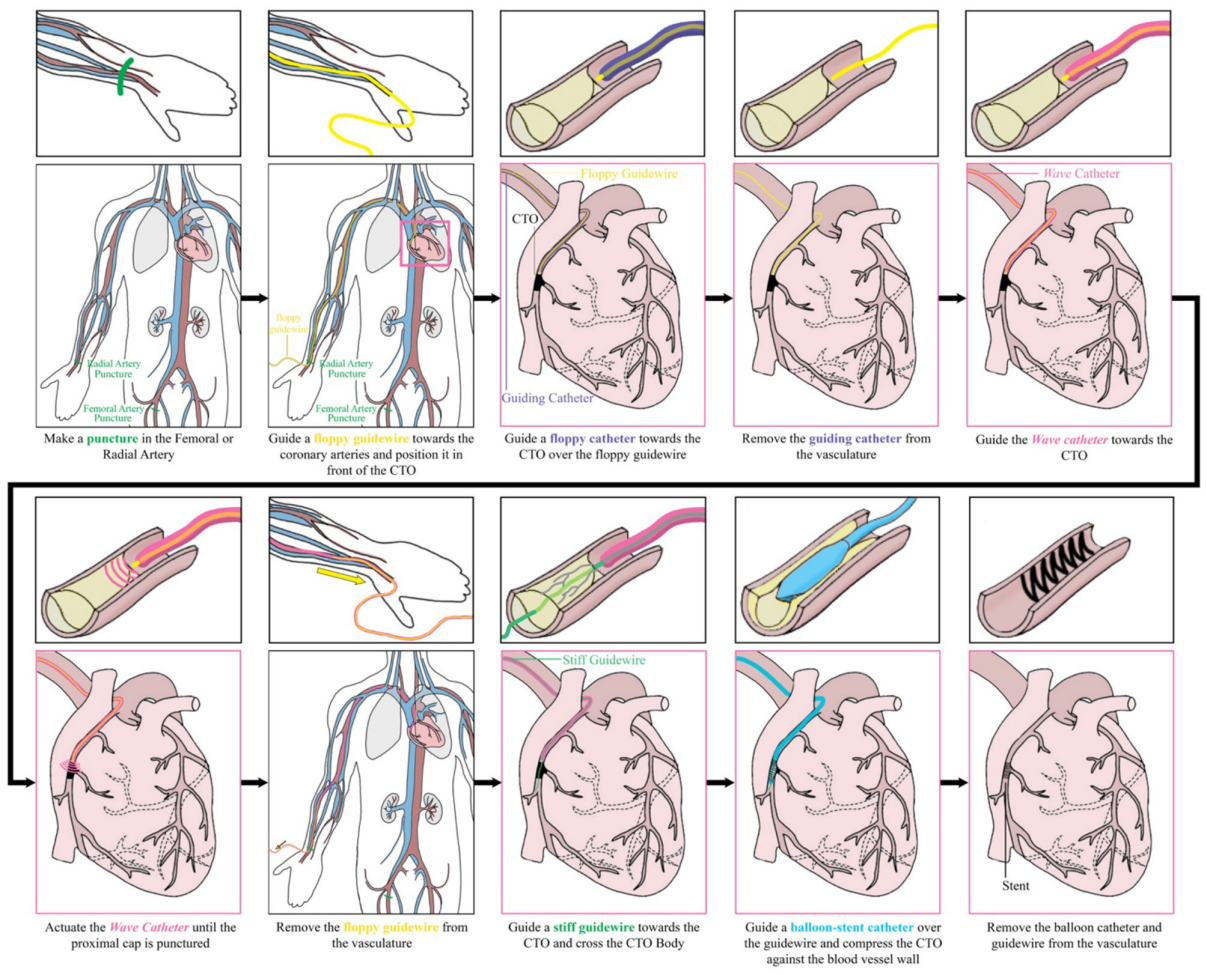
Performing a percutaneous coronary intervention with the *Wave* catheter: intended procedure.

In Figure 15, the intended procedure using the *Wave* catheter during PCI is illustrated. First, the interventionist inserts a floppy guidewire in the vasculature and guides it toward the occlusion under X-ray guidance. Subsequently, a floppy catheter is guided over the guidewires toward the occlusion under X-ray guidance, after which the floppy guidewire is removed from the vasculature. The floppy guidewire is replaced by a support guidewire and, in turn, the floppy catheter is exchanged with a support catheter. At this stage, the interventionist attempts to cross the occlusion with the stiff guidewire. If this step is unsuccessful, the *Wave* catheter can be used. First, the *Wave* catheter is filled with saline until saline is dripping from the distal end of the catheter using the inflator. Subsequently, the interventionist removes the support catheter and slides the distal end of the *Wave* catheter over the support guidewire until the distal end is located right in front of the occlusion. The position of the *Wave* catheter is confirmed using X-ray imaging. Using the handle, the interventionist actuates the *Wave* catheter until a pathway is created. Once the pathway is created, the *Wave* catheter is retracted and handed to the scrub nurse, who places the *Wave* catheter on the instrument table. The stiff guidewire is now used to serve as a guide for the balloon catheter, which is used to compress the plaque material against the blood vessel wall. Finally, the guidewire and balloon catheter are removed from the body and the incision is closed. At the end of the procedure the *Wave* catheter is disposed according to standard hospital practices for biohazard control.

Next to the application in PCI of CTOs, the *Wave* catheter could be used in a variety of other medical fields and applications in which large forces need to exerted through long and slender shafts. The use of an open-ended catheter for fragmenting brittle tissues, could be beneficial in the field of urology for the treatment of kidney, bladder, or gallstones, and orthopedics to penetrate bone or cartilage. Furthermore, the *Wave* catheter could be fitted with a sharp tip to allow for effective tissue resection in cardiac interventions in the heart, such as lead placement and biopsy procedures. Finally, the ability to apply large forces through a flexible tube can also be interested for steerable devices during laparoscopy, for example when precise resection of tissues is required.

### Limitations

This study is subjected to some limitations. In this study, we have developed the *Wave* catheter and have tested in on CTO phantom models made out of a gelatine-HA mixture. Unfortunately, we were unable to test the *Wave* catheter *in-vivo*. Furthermore, only initial insights in the performance behavior of the *Wave* catheter are provided by the limited amount of measurement repetitions that were performed. A larger sample size is needed to provide highly validated results and draw definite conclusions. Furthermore, the peak force of 9.0 ± 0.2 N we were able to achieve in our handheld device is significantly lower than the 40.5 N we were able to achieve previously (21). This is mainly due to two main reasons: (1) the size of the previously used catheter was bigger (6F) than in our current design (5F), and (2) the force applied on the input side is lower in our newly developed design due to the size restraints of the handle. Even though the peak force is significantly lower, the ability to exert 9.0 ± 0.2 N onto target tissues is unparalleled by any other catheter today. With further development of the catheter shaft and the integration of a more powerful solenoid, we feel that higher forces can be achieved still.

### Future Work and Recommendations

Future developments of this catheter should focus on investigating the behavior of the pressure wave inside the catheter shaft and at the tip of the catheter. This could include improving the COMSOL model in which the full shaft geometry is incorporated, experimental work on visualizing the traveling pressure wave in the medium, and *in-vivo* experiments using the catheter. In our current COMSOL model, the catheter is modeled as a cylindrical tube manufactured out of a single polymeric material. However, the developed and commercially available cardiovascular catheters consist of multiple layers: (1) an outer nylon elastomer jacket which provides flexibility and support, (2) a middle layer consisting of a double wired stainless steel braid and coil which stiffens the catheter to provide strength, buckling resistance and allows for easy torqueing, and (3) an inner layer made of PTFE. In future, we recommend incorporating these layers in the COMSOL model to investigate the most optimal configuration for transferring translational momentum through the flexible catheter shaft. Furthermore, the use and effect of different tip shapes and impulse characteristics, such as high frequency impulses, should be researched. Depending on the specific application the catheter will be used for, a different tip shape and impulse characteristic might be preferred. Finally, the implications and applicability of the *Wave* catheter in a clinical setting should be investigated further; first in an animal model and later in a clinical trial. These experiments will give definitive answers on the use of a high-force impulse to cross CTOs and the effect of vessel tortuosity. Even though using a phantom allowed for consistent and repetitive testing of the *Wave* catheter, the phantom did not incorporate blood vessel wall elasticity or take the effect of vessel tortuosity into account. Higher vessel tortuosity might result in significantly higher crossing times, due to higher energy losses in the system. Also, using an open-ended blunt tip, which is beneficial for atraumatic navigation toward the CTO, can result in fluid leakage during insertion, use, and retraction. Even though we are confident that fluid leaking from the catheter should not be harmful to the patient, as we intend to use biocompatible fluids, such as saline, and catheters are already used to inject fluids, such as contrast agents, into the blood stream, the clinical effects should be investigated in future. Additionally, the bending stiffness of ~105·10^−5^ Nm^2^ increases the risk of blood vessel wall perforation. We suggest to add a steerable section to the tip, similar to the ones described in (29, 30), to prevent potential harm to the blood vessel wall in future.

## Conclusion

We have developed an innovative 5F cardiac catheter (*L* = 1,000 mm), called the *Wave catheter*, that allows for applying high forces up to 9.0 ± 0.2 N on target tissues, such as CTOs, without buckling. The catheter uses high-speed pressure waves to transfer high-force impulses through a slender flexible shaft consisted of a flat wire coil, a double braid, and a nylon outer coating. The handle allows for single-handed operation of the catheter with easy adjusting of the input impulse characteristic, including frequency, and easy connection of an off-the-shelf inflator for catheter filling. In a proof-of-principle experiment, we illustrated that the *Wave* catheter was able to penetrate the CTO phantom model. The phantom model was manufactured out of HA and gelatin and was made up of two specific regions representing the CTO proximal cap and body. It was found that the time until puncture decreased from 80 ± 5.4 s to 7.8 ± 0.4 s, for a stroke frequency of 1–10 Hz, respectively. The number of strikes until puncture was approximately constant at 80 ± 5.4, 76.7 ± 2.6, and 77.7 ± 3.9 for the different stroke frequencies. These results show that using a hydraulic pressure wave to deliver high force impulses through long and slender shaft has real potential for use in medical applications, especially with further miniaturization of surgical equipment in the near future.

## Data Availability Statement

The raw data supporting the conclusions of this article will be made available by the authors upon reasonable request.

## Author Contributions

AS writing of the article. SS and JS development of COMSOL model. AS, ML, RvS, and JS design of the prototype. RvS manufacturing of the prototype. ML and RvS performing the experiments. ML, RvS, and JS critical revision of the article. All authors contributed to the article and approved the submitted version.

## Funding

This publication is part of the project *Development of the Wave Catheter for Crossing Chronic Total Occlusions* of the research program NWO Demonstrator which was financed by the Dutch Research Council (NWO) (Grant number: 16104).

## Conflict of Interest

The authors declare that the research was conducted in the absence of any commercial or financial relationships that could be construed as a potential conflict of interest.

## Publisher's Note

All claims expressed in this article are solely those of the authors and do not necessarily represent those of their affiliated organizations, or those of the publisher, the editors and the reviewers. Any product that may be evaluated in this article, or claim that may be made by its manufacturer, is not guaranteed or endorsed by the publisher.

## References

[1] Mouret P. How I developed laparoscopic cholecystectomy. Ann Acad Med Singapore. (1996) 25:744–7. 8924020

[2] Furukawa YMS Ozawa G Wakabayashi M Kitajima T. The revolution of computer-aided surgery–the dawn of robotic surgery. Min Invasive Ther Allied Technol. (2001) 10:283–8. 10.1080/136457001753337320 16754030

[3] Polychronidis A Laftsidis P Bounovas A Simopoulos C. Twenty years of laparoscopic cholecystectomy: Philippe Mouret—March 17, 1987. J Soc Laparoendosc Surg. (2008) 12:109. 18402752 PMC3016026

[4] Vitiello V Lee S-L Cundy TP Yang G-Z. Emerging robotic platforms for minimally invasive surgery. IEEE Rev Biomed Eng. (2012) 6:111–26. 10.1109/RBME.2012.2236311 23288354

[5] Jaffray B. Minimally invasive surgery. Arch Dis Child. (2005) 90:537–42. 10.1136/adc.2004.062760 15851444 PMC1720379

[6] Sakes A Dodou D Breedveld P. Buckling prevention strategies in nature as inspiration for improving percutaneous instruments: a review. Bioinspir Biomim. (2016) 11:021001. 10.1088/1748-3190/11/2/021001 26891469

[7] Euler L. Principes généraux de l'état d'équilibre des fluides. Mémoires de l'académie des sciences de Berlin. (1757). p. 217–73.

[8] Karagiozova D Alves M. Dynamic elastic-plastic buckling of structural elements: a review. Appl Mech Rev. (2008) 61:040803. 10.1115/1.2939481

[9] Kuzkin VA. Structural model for the dynamic buckling of a column under constant rate compression. arXiv preprint arXiv. (2015). 1506.00427.

[10] Heverly M Dupont P Triedman J. Trajectory optimization for dynamic needle insertion. In: Proceedings of the 2005 IEEE International Conference on Robotics and Automation: IEEE. (2005). p. 1646–51.

[11] Jelínek F Smit G Breedveld P. Bioinspired spring-loaded biopsy harvester—experimental prototype design and feasibility tests. J Med Device. (2014) 8:015002. 10.1115/1.4026449

[12] Thind A Strauss B Teitelbaum A Karshafian R Ladouceur M Whyne C. A novel method for the measurement of proximal fibrous cap puncture force in chronic total occlusions: the effect of increasing age. EuroIntervention. (2011) 6:997–1002. 10.4244/EIJV6I8A172 21330249

[13] Kinoshita I Katoh O Nariyama J Otsuji S Tateyama H Kobayashi T. Coronary angioplasty of chronic total occlusions with bridging collateral vessels: immediate and follow-up outcome from a large single-center experience. J Am Coll Cardiol. (1995) 26:409–15. 10.1016/0735-1097(95)80015-9 7608443

[14] Sakes A Regar E Dankelman J Breedveld P. Crossing total occlusions: navigating towards recanalization. Cardiovasc Eng Technol. (2016) 7:103–17. 10.1007/s13239-016-0255-0 26831298 PMC4858560

[15] Sakes A Regar E Dankelman J Breedveld P. Treating total occlusions: applying force for recanalization. IEEE Rev Biomed Eng. (2016) 9:192–207. 10.1109/RBME.2016.2580218 27323373

[16] Waksman R Saito S. Chronic Total Occlusions: A Guide to Recanalization. Chichester: John Wiley & Sons (2013).

[17] Vemmou E Nikolakopoulos I Xenogiannis I Megaly M Hall A Wang Y. Recent advances in microcatheter technology for the treatment of chronic total occlusions. Expert Rev Med Devices. (2019) 16:267–73. 10.1080/17434440.2019.1602039 30929525

[18] Kinnaird T Gallagher S Cockburn J Sirker A Ludman P De Belder M. Procedural success and outcomes with increasing use of enabling strategies for chronic total occlusion intervention. Circ Cardiovasc Intervent. (2018) 11:e006436. 10.1161/CIRCINTERVENTIONS.118.006436 30354634

[19] Xenogiannis I Karmpaliotis D Alaswad K Jaffer FA Yeh RW Patel M. Usefulness of atherectomy in chronic total occlusion interventions (from the PROGRESS-CTO registry). Am J Cardiol. (2019) 123:1422–8. 10.1016/j.amjcard.2019.01.054 30798947

[20] Walsh SJ Cosgrove C Spratt JC Hanratty CG. A technical focus on antegrade dissection and re-entry for coronary chronic total occlusions: a practice update for 2019. Korean Circ J. (2019) 49:559–67. 10.4070/kcj.2019.0160 31243929 PMC6597452

[21] Sakes A Nicolai T Karapanagiotis J Breedveld P Spronck JW. Crossing Total Occlusions using a hydraulic pressure wave: a feasibility study. Biomed Phys Eng Express. (2018) 4:055019. 10.1088/2057-1976/aad44a

[22] Ghidaoui MS Zhao M Mcinnis DA Axworthy DH. A review of water hammer theory and practice. Appl Mech Rev. (2005) 58:49–76. 10.1115/1.1828050

[23] Korson L Drost-Hansen W Millero FJ. Viscosity of water at various temperatures. J Phys Chem. (1969) 73:34–9. 10.1021/j100721a006

[24] Qasem N Generous MM Qureshi B Zubair S. A comprehensive review of saline water correlations and data: part II—thermophysical properties. Arab J Sci Eng. (2021). 10.1007/s13369-020-05020-5

[25] Jeroudi OM Alomar ME Michael TT Sabbagh AE Patel VG Mogabgab O. Prevalence and management of coronary chronic total occlusions in a tertiary Veterans Affairs hospital. Catheter Cardiovasc Interv. (2014) 84:637–43. 10.1002/ccd.25264 24142769 PMC3992199

[26] Stone GW Kandzari DE Mehran R Colombo A Schwartz RS Bailey S. Percutaneous recanalization of chronically occluded coronary arteries. Circulation. (2005) 112:2364–72. 10.1161/CIRCULATIONAHA.104.481283 16216980

[27] Barrett SRH Sutcliffe MPF Howarth S Li ZY Gillard JH. Experimental measurement of the mechanical properties of carotid atherothrombotic plaque fibrous cap. J Biomech. (2009) 42:1650–5. 10.1016/j.jbiomech.2009.04.025 19464014

[28] Cheng NS. Formula for the viscosity of a glycerol– water mixture. Ind Eng Chem Res. (2008) 47:3285–8. 10.1021/ie071349z

[29] Sakes A Ali A Janjic J Breedveld P. Novel miniature tip design for enhancing dexterity in minimally invasive surgery. J Med Device. (2018) 12:035002. 10.1115/1.4040636

[30] Ali A Sakes A Arkenbout EA Henselmans P Van Starkenburg R Szili-Torok T. Catheter steering in interventional cardiology: mechanical analysis and novel solution. Proc Inst Mech Eng H. (2019) 233:1207–18. 10.1177/0954411919877709 31580205 PMC6859597

